# Steroid hormone regulation of prostate-specific antigen gene expression in breast cancer.

**DOI:** 10.1038/bjc.1997.101

**Published:** 1997

**Authors:** N. Zarghami, L. Grass, E. P. Diamandis

**Affiliations:** Department of Pathology and Laboratory Medicine, Mount Sinai Hospital, Toronto, Ontario, Canada.

## Abstract

**Images:**


British Journal of Cancer (1997) 75(4), 579–588
© 1997 Cancer Research Campaign

### Steroid hormone regulation of prostate-specific antigen gene expression in breast cancer

**N Zarghami[1,2], L Grass[1] and EP Diamandis[1,2]**

[1]Department of Pathology and Laboratory Medicine, Mount Sinai Hospital, 600 University Avenue, Toronto, Ontario M5G 1X5, Canada;
[2]Department of Clinical Biochemistry, University of Toronto, 100 College Street, Toronto, Ontario M5G 1L5, Canada

**Summary** We have recently reported that about 30–40% of female breast tumours produce prostate-specific antigen (PSA) and that PSA production is associated with the presence of oestrogen (ER) and progesterone (PR) receptors. We have now developed a tissue culture system to study the regulation of the *PSA* gene in breast cancer. The breast carcinoma cell line T-47D produces PSA when stimulated by androgens, progestins and glucocorticoids/mineralocorticoids but not oestrogens. PSA mRNA appears approximately 2 h after stimulation; PSA protein appears after 4–8 h. Among 38 compounds tested, only androgens and progestins were able to stimulate PSA production at concentrations below $10^{-9}$ M. Evidence that the progesterone and androgen receptors can regulate the *PSA* gene independently was provided as follows: (a) the progestin norgestimate, which does not bind to the androgen receptor, up-regulates the *PSA* gene at concentrations as low as $10^{-10}$ M; (b) triamicinolone acetonide, which does not bind to the androgen receptor (AR) but binds to the PR, acts similarly to norgestimate; (c) the antiandrogen cyproterone acetate, which blocks the androgen receptor but has progestational activity, up-regulates the *PSA* gene at concentrations as low as $10^{-10}$ M; (d) the antiprogestin mifepristone completely blocks the stimulation of the specific progestin norgestimate. Our tissue culture system identified androgen – progestin agonist activities of 17α-ethinyloestradiol, the antioestrogen RU56, 187 and the antiprogestin mifepristone. Our data suggest that the expression of the *PSA* gene in the female breast is under the control of androgens and progestins. Our tissue culture system is a highly sensitive in vitro method for evaluating the biological activity of candidate compounds having agonist and antagonist steroid hormone activity.

**Keywords:** prostate-specific antigen; steroid hormone receptors; breast cancer; *PSA* gene regulation; anti-cancer agents; progestins; antiprogestins; androgens; antiandrogens

Prostate-specific antigen (PSA) is a 33-kDa serine protease produced at high concentrations by prostatic epithelial cells and secreted into the seminal plasma. PSA production in the prostate is regulated by androgens through the action of the androgen receptor. Until recently, PSA was thought to be a prostatic tissue-specific protein that is not expressed in any other tissue in men or women. We have shown that PSA production occurs in the female breast and some other tissues in both men and women (Diamandis and Yu, 1995). Normal, benign and malignant breast tissue produces PSA (Yu et al, 1995*a*); many malignant breast tumours lose their ability to produce PSA. PSA appears to be a prognostic indicator in breast cancer (Yu et al, 1995*b*). In our previous studies, we have found that there is a close association between PSA presence in breast tumours and presence of both oestrogen and progesterone receptors; this association was stronger between PSA and progesterone receptors (Yu et al, 1994*a*).

In order to investigate the mechanism of *PSA* gene regulation in the breast, we have developed a tissue culture system which reproduces in vitro the phenomenon of PSA production by breast cells. The steroid hormone receptor-positive breast carcinoma cell line T-47D does not produce detectable PSA when cultured in media devoid of steroid hormones (Yu et al, 1994*b*). When stimulated by steroid hormones, this cell line produces PSA in a dose-dependent manner. We have used this system to study the kinetics of PSA production, the dose–response of PSA production by various steroid hormones and the blocking effect of anti-hormones. We provide evidence that the *PSA* gene in this cell line is regulated by androgens and progestins.

*Received 29 April 1996*
*Revised 12 August 1996*
*Accepted 29 August 1996*

*Correspondence to:* EP Diamandis, Department of Pathology and Laboratory Medicine, Mount Sinai Hospital, 600 University Ave., Toronto, Ontario M5G 1X5, Canada

### METHODS

### Compounds

All steroidal and non-steroidal compounds used in this study were obtained from Sigma Chemical, St Louis, MO, USA, except for the following: ICI 102, 780 and casodex (ICI 176, 334) (Zeneca Pharma, Mississauga, ON, Canada); RU58, 668, RU54, 876, RU56, 187, nilutamide (Anandron, RU23, 908) and mifepristone (RU486, RU38, 486) (Roussel-UCLAF, Romainville, France). Hydroxyflutamide was a gift from Dr Donna Peehl, Stanford University. Stock solutions ($10^{-2}$ or $10^{-3}$ M) were prepared in absolute ethanol. More dilute solutions were also prepared in the same solvent.

### Stimulation experiments

The T-47D breast carcinoma cell line was obtained from the American Type Culture Collection (ATCC), Rockville, MD, USA. This cell line is positive for oestrogen, progesterone, androgen and glucocorticoid receptors, although the amounts of each receptor are disputed (Glover and Darbre, 1989; Nordeen et al, 1989). T-47D cells were cultured in RPMI media (Gibco BRL, Gaithersburg, MD, USA) supplemented with glutamine (200 mmol $l^{-1}$), bovine insulin (10 mg $l^{-1}$), fetal bovine serum (10%), antibiotics (penicillin, streptomycin) and antimycotics (amphotericin B). The cells were cultured to near confluency in plastic culture flasks and then transferred to phenol red-free media containing 10% charcoal-stripped fetal bovine serum with antibiotics/antimycotics. Phenol red-free media were used as phenol red was previously found to have weak oestrogenic activity (Berthois et al, 1986) and charcoal-stripped fetal bovine serum is devoid of steroid hormones.

The T-47D cells were then aliquoted into 24-well tissue culture plates (Corning no. 25820) and cultured to confluency with change in media at 3 days. Stimulations were carried out with confluent cells containing 2 ml of phenol red-free media with 10% charcoal-stripped fetal calf serum and antibiotics/antimycotics. Stimulation was initiated by adding 2 µl of each steroid dissolved in 100% ethanol and incubating for a certain period of time (usually up to 8 days). Tissue culture supernatants (~150 µl) were removed for PSA analysis at days 3, 5 and 8. Slight modifications of this protocol were introduced as necessary. Appropriate multiple positive and negative controls (only alcohol added) were included in each experiment. Wells with microbial contamination were excluded from the data analysis.

We also used the cell lines SAOS (osteosarcoma; provided by Dr M Grynpas, Mount Sinai Hospital, Toronto, Canada) and BG-1 (ovarian carcinoma; provided by Dr H Rocheford, INSERM, Montpellier, France) as well as the steroid hormone receptor-negative cell line BT-20 (breast carcinoma; obtained from ATCC). These cell lines were treated similarly to the T-47D cells.

### Dose–response experiments

For dose–response experiments, we followed the same protocol as for stimulation, but steroids (2 µl per well) were added at various concentrations. Steroid dilution in each well was 1000-fold in all experiments as we added 2 µl of steroid solution in 2 ml of tissue culture medium. The final concentration of each steroid was used for data interpretation.

### Blocking experiments

Blocking experiments were performed by simultaneously checking for the following possibilities: (a) stimulation by the blocker alone at a final concentration of $10^{-8}$ M; (b) stimulation by the stimulating steroid alone at a final concentration of $10^{-9}$ M; (c) adding the blocker to the cells at a final concentration of $10^{-8}$ M, incubating for 1 h and then adding the stimulant at a concentration of $10^{-9}$ M; (d) including controls with ethanol only (negative controls). This protocol allows for a direct comparison of the stimulating activity of either the blocker or the stimulant and the effect of the blocker on the ability of the stimulant to induce PSA expression when the blocker is allowed to bind to the receptors at tenfold higher concentrations for 1 h before the addition of the stimulant.

### Kinetic experiments

The kinetics of PSA production by T-47D cells was studied as follows: confluent T-47D cells were stimulated with norgestrel at a final concentration of $10^{-8}$ M, and the cells were harvested along with tissue culture supernatants at 1, 2, 4, 8, 24 and 48 h. Control cells were harvested at 48 h without any stimulation (ethanol added only). The tissue culture supernatants and a portion of the cells were used for PSA protein analysis; another portion of the cells was used to extract total RNA for polymerase chain reaction (PCR) analysis of PSA mRNA.

### Lysis procedure

The cell pellets were lysed for 30 min on ice with 1 ml of lysis buffer. Lysis buffer was 50 mmol $l^{-1}$ Tris, pH 8.0 containing 150 mmol $l^{-1}$ sodium chloride, 5 mmol $l^{-1}$ ethylenediaminetetraacetic acid (EDTA), 10 g $l^{-1}$ nonidet NP-40 surfactant, 1 mmol $l^{-1}$ phenylmethylsulphonyl fluoride and 1 mg $l^{-1}$ each of aprotinin and leupeptin as proteinase inhibitors. The lysate was centrifuged at 15 000 **g** at 4°C for 30 min, the supernatant was collected and immediately assayed for PSA and total protein.

### Measurement of PSA

PSA was measured with a highly sensitive immunofluorometric procedure described in detail elsewhere (Ferguson et al, 1996). This assay can measure PSA at levels of 10 ng $l^{-1}$ or higher (up to 10 000 ng $l^{-1}$) with a precision of <10%. All assays were performed in duplicate. Tissue culture supernatants were measured undiluted using 50 µl aliquots per assay. T-47D cells were detached by trypsin-EDTA treatment, washed in phosphate-buffered saline and then lysed in lysis buffer before analysis for PSA.

### Measurement of total protein

Cell lysates were quantified for total protein using the bicinchoninic acid (BCA) total protein method commercially available by Pierce Chemical, Rockford, IL, USA.

### Extraction of total RNA

Total RNA from T-47D cells was extracted using a commercial reagent, TRIzol (Gibco BRL). The quality and quantity of the extracted RNA was checked by spectrophotometric measurements at 260 and 280 nm.

### Reverse transcription

One microgram of total RNA was reverse transcribed using oligo dT primers and Superscript II reverse transcriptase (Gibco BRL). Briefly, RNA and oligo (dT) primers (500 ng) were first denatured for 10 min at 70°C, chilled on ice for 1 min and then incubated for 1 h at 42°C in a 20-µl reaction mixture containing 1 × PCR buffer, 2.5 mM magnesium chloride, 1 mM deoxynucleoside triphosphates, 10 mM dithiothreitol and 200 units of Superscript II reverse transcriptase. The reaction was terminated by heating for 15 min at 70°C. Template RNA was digested by incubation with RNAase H for 20 min at 37°C.

### Oligonucleotide primers

We have used two oligonucleotides to amplify the cDNA of PSA with the polymerase chain reaction (PCR). These were originally proposed by Deguchi et al (1993) and they have the following sequences:

© Cancer Research Campaign 1997

**Figure 1** RT-PCR of PSA mRNA extracted from T-47D cells. (**A**) Ethidium bromide-stained agarose gel; (**B**) The gel was Southern-transferred and the PCR product, in which digoxigenin-dUTP was incorporated during PCR, was detected with anti-digoxigenin antibodies and chemiluminescence. Lanes 1 and 7: T-47D cells were stimulated with absolute alcohol (solvent) and mRNA extracted at the beginning (0 time) or at the end (48 h) of the experiment respectively. Lanes 2, 3,4,5,6 and 8. The T-47D cells were stimulated with $10^{-8}$ M norgestrel once and mRNA extracted after 1 h (lane 2), 2 h (lane 3), 4 h (lane 4), 8 h (lane 5), 24 h (lane 6) and 48 h (lane 8). MW markers, molecular weight markers. The PCR product is 754 bp in size. Actin RT-PCR was performed in all cDNAs from lanes 1–8 and it was positive in all cases (data not shown)

PSA-1: 5′-TGC-GCA-AGT-TCA-CCC-TCA-3′
PSA-2: 5′-CCC-TCT-CCT-TAC-TTC-ATC-C-3′

These primers amplify a 754-bp fragment of PSA cDNA. Actin primers were used as controls; their sequences have been described previously (Okazaki, 1992). Actin primers amplify a 372-bp fragment of actin cDNA.

### PCR procedure

One microlitre of cDNA was added to 49 μl of PCR mix containing 1 × PCR buffer (Boehringer Mannheim), 2.5 mM magnesium chloride, 500 nM of PCR primers, 200 μM of deoxynucleoside triphosphates and 1.25 units of *Taq* DNA polymerase (Boehringer Mannheim). PCR was performed for 30 cycles according to the following programme on the Perkin-Elmer 2400 thermal cycler: 94°C for 30 s (5 min for the first cycle), 60°C for 30 s: and 72°C for 30 s (7 min for the last extension). Actin cDNA was amplified from 1 μl of the cDNA preparation under the same conditions used for PSA cDNA. Fifteen microlitres of each PCR reaction were electrophoresed on 2% agarose gels and visualized by ethidium bromide staining. In other experiments, during the PCR reaction, we incorporated digoxigenin-11-dUTP and detected the PCR product after Southern transfer to nylon membranes and

**Figure 2** Appearance of PSA mRNA and PSA protein intracellularly (◘) or in the tissue culture supernatant (◆) following stimulation of T-47D cells with $10^{-8}$ M norgestrel. The post-stimulation time of first appearance of PSA mRNA is 2 h, of intracellular PSA protein is 4 h and of PSA protein in tissue culture supernatant is 8 h. Cells not stimulated at all (data not shown) or stimulated with ethanol did not produce PSA mRNA or protein during the 48 h duration of this experiment. No PSA mRNA or protein was seen in norgestrel-stimulated or unstimulated BT-20 breast carcinoma cell lines (data not shown)

probing with anti-digoxigenin antibodies conjugated to alkaline phosphatase (ALP). ALP activity was detected with chemiluminescence. This method is about 50 times more sensitive than ethidium bromide staining in detecting the PCR products. Digoxigenin-11-dUTP (Boehringer) was added in the PCR mix at a concentration of 0.7 μM.

### Sequencing of PCR products

Total RNA was extracted from norgestrel-stimulated T-47D cells, reverse transcribed and amplified as described above. The PCR product was sequenced with the Thermo Sequenase fluorescent labelled primer cycle sequencing kit (Amersham International, Buckinghamshire, UK) following the recommendations of the manufacturer. Our sequencing primers, labelled at the 5′-end with CY.5 fluorescent dye, had the following sequences: PSA-S1: 5′-AAGGTGACCAAGTTCATG-3′ (binds 19 bases internally from PCR primer PSA-1). PSA-S2: 5′-CCATCCCATGCCAAAGGA-3′ (binds 19 bases internally from PCR primer PSA-2). All sequencing reactions were loaded on the ALF Express automatic sequencer (Pharmacia Biotech, Uppsala, Sweden).

### RESULTS

The breast carcinoma cell line T-47D was cultured in the absence of any stimulating steroid and in the presence of the stimulating steroid norgestrel at a concentration of $10^{-8}$ M. The appearance of PSA mRNA was monitored with reverse transcription–polymerase chain reaction (RT-PCR). The appearance of PSA protein inside and outside of the cell (secreted protein) was monitored by the immunoassay procedure. The results are summarized in Figures 1 and 2.

PSA mRNA is undetectable in either unstimulated cells, cells stimulated with ethanol for up to 48 h or cells stimulated with norgestrel after 1 h post-stimulation. PSA mRNA becomes detectable in the norgestrel-stimulated cells at 2 h, its concentration increases at 4 h and it persists for at least 48 h (Figure 1). However,

© Cancer Research Campaign 1997

**Figure 3** Representative chromatogram of an 82-nucleotide region of the PSA cDNA sequence from norgestrel-stimulated T-47D cells. For details see text

| | | | | | |
|---|---|---|---|---|---|
| 1 | GCTC**G**GGTGA | TTCTGGGGGC | CCACTTGTCT | GTAATGGTGT | GCTTCAAGGT |
| 51 | ATCACGTCAT | GGGGCAGTGA | ACCATGTGCC | CTGCCCGAAA | GGCCTTCCCT |
| 101 | GTACACCAAG | GTGGTGCATT | ACCGGAAGTG | GATCAAGGAC | ACCATCGTGG |
| 151 | CCAACCCCTG | **A**GCACCCCTA | TCAACTCCCT | ATTGTAGTAA | ACTTGGAACC |
| 201 | TTGGAAATGA | CCAGGCCAAG | ACTCAGGCCT | CCCCAGTTCT | ACTGACCTTT |
| 251 | GTCCTTAGGT | GTGAGGTCCA | GGGTTGCTAG | GAAAAGAAAT | CAGCAGACAC |
| 301 | AGGTGTAGAC | CAGAGTGTTT | CTTAAATGGT | GTAATTTTGT | CCTCTCTGTG |
| 351 | TCCTGGGGAA | TACTGGCCAT | GCCTGGAGAC | ATATCACTCA | ATTTCTCTGA |
| 401 | GGACACAGAT | AGGATGGGGT | GTCTGTGTTA | TTTGTGGGGT | ACAGAGATGA |
| 451 | AAGAGGGGTG | GGATCCACAC | TGAGAGAGTG | GAGAGTGACA | TGTGCTGGAC |
| 501 | ACTGTCCATG | AAGCACTGAG | CAGAAGCTGG | AGGCACAACG | CACCAGACAC |
| 551 | TCACAGCAAG | GATGGAGCTG | AAAACATAAC | CCACTCTGTC | CTGGAGGCAC |
| 601 | TGGGAAGCCT | AGAGAA | | | |

**Figure 4** Complete sequence of the 616 nucleotide region of the PSA cDNA sequence from norgestrel-stimulated T-47D cells. For discussion, see text. Bold and underline indicate end of exon 4 and exon 5 respectively this test is semiquantitative. Quantitative information was provided by protein data. PSA protein is first detected in the cell cytoplasm 4 h after stimulation by Norgestrel and accumulates over the 48-h study period. PSA secreted into the culture medium is first detected at 8 h, and its concentration increases rapidly with time (Figure 2).

An identical experiment was performed using the steroid hormone receptor-negative breast carcinoma cell line BT-20. This cell line did not produce any detectable PSA mRNA or protein after stimulation with norgestrel at the indicated time periods (data not shown).

The identity of the PCR product was verified by complete sequencing of both strands. Partial sequencing data are shown in Figure 3. The entire sequence is shown in Figure 4. The sequence, spanning 616 nucleotides from exons 4, 5 and the 3′-untranslated region, is >99% homologous to the published sequence of PSA cDNA or genomic DNA. We found 100% homology with the sequence published by Lundwall (1989), Digby et al (1989) and Klobeck et al (1989).

There is one base difference (A to G) at position 439 (3′-untranslated region) of our sequence and the sequence published by Lundwall and Lilja (1987), Shultz et al (1988), Henttu and Vihko (1989) and Riegman et al (1988). Also, at position 419 (3′-untranslated region), we and others have identified G but a few other investigators have identified T. These differences are likely polymorphisms.

Comparison of our 616 nucleotide sequence with the sequence of human glandular kallikrein gene revealed only about 80% homology. These data confirm that the mRNA isolated from norgestrel-stimulated T-47D cells is indeed the PSA mRNA.

In order to establish which of the compounds shown in Table 1 act as *PSA* gene regulators, we stimulated T-47D cells and measured PSA in the tissue culture supernatant at 3, 5 and 8 days after single stimulation at a compound concentration of $10^{-7}$M. The compounds were then qualitatively categorized into three groups: non-stimulators, weak stimulators and strong stimulators. For this classification, we arbitrarily selected a supernatant PSA concentration at 8 days of >200 ng $l^{-1}$, 10–200 ng $l^{-1}$ or <10 ng $l^{-1}$ for strong, weak and non-stimulators respectively. Some stimulation data are shown graphically in Figure 5; detailed data are summarized in Table 1.

Dose–response experiments were designed to determine the lowest concentration of stimulating steroids which could still

© Cancer Research Campaign 1997

**Table 1** Regulation of PSA gene expression by various compounds

| Compound | Major biological activity | PSA production at $10^{-7}$M | Lowest concentration for response (M) |
|---|---|---|---|
| Testosterone | Androgen | Strong[a] | $10^{-10}$ |
| Dihydroandrosterone | Androgen | Strong | $\leq 10^{-11}$ |
| Androsterone | Androgen | Strong | $10^{-10}$ |
| R1881 (Methyltrienolone) | Androgen | Strong | ND |
| R5020 | Androgen/progestin | Strong | ND |
| Dihydrotestosterone | Androgen | Strong | $\leq 10^{-11}$ |
| Dihydroisoandrosterone sulphate (DHEA-$SO_4$) | Androgen metabolite | Nothing[a] | – |
| Oestrone | Oestrogen | Nothing | – |
| Oestriol | Oestrogen metabolite | Nothing | – |
| 17 α-Ethynyloestradiol | Oestrogen | Weak[a] | $10^{-8}$ |
| β-Oestradiol | Oestrogen | Nothing | – |
| Corticosterone | Glucocorticoid | Weak | $10^{-7}$ |
| Hydrocortisone | Glucocorticoid | Nothing | – |
| Betamethasone 17-valerate | Glucocorticoid | Strong | ND |
| Dexamethasone | Glucocorticoid | Strong | $10^{-8}$ |
| Prednisone | Glucocorticoid | Nothing | – |
| 17α-Hydroxyprogesterone | Progesterone precursor | Nothing | – |
| Progesterone | Progestin | Weak | $10^{-10}$ |
| Norethynodrel | Progestin | Strong | ND |
| Norethidrone | Progestin | Strong | ND |
| Norgestrel | Progestin | Strong | $\leq 10^{-11}$ |
| Depo-provera | Progestin | Strong | $\leq 10^{-11}$ |
| Norgestimate | Progestin | Strong | $10^{-10}$ |
| Aldosterone | Mineralocorticoid | Weak | $10^{-8}$ |
| Triamicinolone acetonide | Progestin/glucocorticoid | Strong | $10^{-10}$ |
| Tamoxifen | Antioestrogen | Nothing | – |
| ICI 182, 780 | Antioestrogen | Nothing | – |
| RU 58, 668 | Antioestrogen | Nothing | – |
| RU 54, 876 | Antioestrogen | Nothing | – |
| Hydroxyflutamide | Antiandrogen | Nothing | – |
| Cyproterone acetate | Antiandrogen/progestin | Strong | $10^{-10}$ |
| Casodex (ICI 176, 334) | Antiandrogen | Nothing | – |
| RU56, 187 | Antiandrogen | Weak | $10^{-8}$ |
| Nilutamide (Anandron) | Antiandrogen | Nothing | – |
| Mifepristone (RU486/RU38, 486) | Antiprogestin | Weak | $10^{-8}$ |
| Cortexolone | Antiglucocorticoid | Weak | $10^{-7}$ |
| Spironolactone | Antimineralocorticoid | Weak | $10^{-7}$ |
| Cholesterol | Steroid hormone precursor | Nothing | – |
| Alcohol | Nothing (solvent) | Nothing | – |

[a]For definition, see text. ND, not done.

**Figure 5** PSA concentration in T-47D cell line tissue culture supernatants after a single $10^{-7}$ M stimulation with various compounds and sampling of the supernatants at 3 (■), 5 (●) and 8 (▲) days after stimulation. The compounds tested were: 1, testosterone; 2, oestrone; 3, vitamin D; 4, corticosterone; 5, dihydroandrosterone; 6, oestriol; 7, 17α-hydroxyprogesterone; 8, androsterone; 9, hydrocortisone; 10, 17α-ethynyloestradiol; 11, norethynodrel; 12, tamoxifen; 13, β-oestradiol; 14, betamethasone-17-valerate; 15, norethidrone; 16, norgestrel; 17, aldosterone; 18, dexamethasone; 19, cholesterol; 20, R1881; 21, R5020; 22, 23, no stimulation; 24, alcohol. In all cases the most dramatic change in PSA concentration occurs in the interval between 3 and 6 days. The strongest stimulators are androgens (1, 5, 8, 20, 21) and progestins (11, 15, 16). More data are given in Table 1

**Figure 6** Dose–response experiments of six representative steroids. For more details and discussion, see text induce production of PSA. Representative dose–response experiments are shown in Figure 6. The lowest concentration of stimulating steroids which could induce PSA production is shown in Table 1. Among the steroids tested, the most potent were two androgens (dihydroandrosterone and dihydrotestosterone) and two synthetic progestins (norgestrel and Depo-provera). These four steroids could induce PSA production at final concentrations down to $10^{-11}$ M. Two other androgens (testosterone and androsterone) and a synthetic progestin (Norgestimate) were potent at concentrations down to $10^{-10}$ M. 17α-Ethinyloestradiol, unlike the other oestrogens tested, was able to regulate PSA production positively, but only at relatively high concentrations ($10^{-7}$–$10^{-8}$ M). Among the glucocorticoids, corticosterone was a weak stimulator whereas betamethazone and dexamethazone were strong stimulators, but only at concentrations $\geq 10^{-8}$ M. Aldosterone was a weak stimulator but the synthetic compound triamicinolone acetonide (TA) was a strong stimulator, acting at low concentrations ($10^{-10}$ M). None of the antioestrogens exhibited stimulatory activity. Among the antiandrogens, some did not induce any PSA production, one was a weak stimulator (RU 56, 187) and one (cyproterone acetate) was a strong stimulator, acting at concentrations as low as $10^{-10}$ M. The antiprogestin mifepristone (RU 38, 486; RU 486) as well as the antiglucocorticoid cortexolone and the antimineralocorticoid spironolactone were weak stimulators, acting at concentrations $10^{-7}$–$10^{-8}$ M. The androgen metabolite dihydroisoandrosterone sulphate and the progesterone precursor 17α-hydroxyprogesterone were inactive. Dose–response experiments with the oestrogens oestrone and oestradiol have shown that these compounds remain inactive with respect to *PSA* gene up-regulation at any concentration between $10^{-7}$ and $10^{-10}$ M.

*© Cancer Research Campaign 1997*

**Table 2** Blocking of PSA production in T-47D cells by various compounds[a]

| Stimulating compound ($10^{-9}$M | Blocking compound ($10^{-8}$M | Blocking of Dihyrotestosterone[b] (%) | Blocking of Norgestrel[b] (%) | Blocking of Norgestimate[b] (%) |
|---|---|---|---|---|
| Dihydrotestosterone | Oestradiol | 50–80 | 9–12 | 0 |
| or | Nilutamide | 20–40 | 0–15 | 0 |
| Norgestrel | RU 56,187 | 85–91 | 17–40 | 0 |
| or | Hydroxyflutamide | 30–33 | 0–20 | 0 |
| Norgestimate | ICI 182, 780 | 0 | 10–20 | 0 |
| | Mifepristone | 70–80 | 90–100 | 100 |
| | Spironolactone | 0 | 0 | 0 |
| | Cortexolone | 0 | 0 | 0 |

[a]For detailed protocol refer to Methods section. [b]Range of three different experiments.

Stimulation experiments were further conducted using the steroid hormone receptor-genomic negative breast carcinoma cell line BT-20 and the steroid hormone receptor-positive cell lines SAOS (osteosarcoma) and BG-1 (ovarian carcinoma). None of the compounds listed in Table 1 was able to induce detectable PSA protein production.

In order to further elucidate the mechanism of regulation of PSA production by steroid hormones, we have conducted blocking experiments. In these studies, we have first treated the T-47D cells with a steroid hormone receptor blocker for 1 h followed by the addition of a tenfold lower concentration of a stimulating steroid. The data are presented in Table 2.

Oestradiol, as well as the antiandrogens nilutamide, RU56, 187 and hydroxyflutamide, was able to block significantly the stimulating action of dihydrotestosterone. Mifepristone was also a potent blocker; no blocking activity was seen among the antioestrogen ICI 182, 780 the antimineralocorticoid spironolactone and the antiglucocorticoid cortexolone.

The stimulatory activity of norgestrel was blocked minimally by oestradiol, nilutamide, hydroxyflutamide and ICI 182, 780 and to a higher degree by RU 56, 187. The most potent blocker of norgestrel's action was the antiprogestin mifepristone (blocking 90–100%).

The stimulatory activity of the highly selective progestin agonist norgestimate was only blocked by the antiprogestin mifepristone (blocking 100%).

### DISCUSSION

The *PSA* gene is known to be regulated by androgens in the male prostate (Henttu et al, 1992; Levine, 1995). The epithelial cells of the prostate gland are rich in AR; some stromal cells also contain AR, as well as the enzyme 5α-reductase, which reduces testosterone to dihydrotestosterone (Levine, 1995). The *PSA* gene has a hormone response element (HRE) to which the activated AR can bind (Klobeck et al, 1989; Riegman et al, 1991; Murtha et al, 1993; Luke and Coffey, 1994). The PSA gene is up-regulated by androgens and androgen agonists and is down-regulated by antiandrogens.

We have recently shown that female breast tissue and breast secretions contain high levels of PSA (Diamandis and Yu, 1995). Although the physiological role of this protein in the female breast is still unknown, we have demonstrated that the presence of PSA is strongly associated with presence of steroid hormone receptors (Yu et al, 1994*a*). We have thus postulated that the *PSA* gene in the female breast is regulated by steroid hormones. In this study, we have developed a tissue culture system to further examine this regulation and study the involvement of the various steroid hormone receptors.

We have first shown that the steroid hormone receptor-positive breast carcinoma cell line T-47D is capable of producing PSA under appropriate stimulation by steroid hormones. T-47D cells, as well as MCF-7 cells, do not produce PSA in the absence of steroid hormones (Yu et al, 1994*b*; Smith et al, 1995). The PSA mRNA produced by T-47D cells is identical to the sequence of PSA mRNA from prostate cells. In contrast, the breast carcinoma cell line BT-20, which is devoid of steroid hormone receptors, did not produce PSA after stimulation by any of the compounds listed in Table 1. We have thus postulated that PSA production by breast cells is dependent on the steroid hormone–steroid hormone receptor system. We further demonstrated that the receptors and hormones are necessary but not sufficient for PSA production. When we stimulated the steroid hormone receptor-positive cell lines SAOS (osteosarcoma) and BG-1 (ovarian carcinoma) with the compounds shown in Table 1, none was able to induce PSA production. The presence of oestrogen and progesterone receptors in these cell lines was confirmed by analysis with established enzyme immunoassay kits (data not shown). Apparently, either post-receptor defects are present in these cell lines, the receptors are defective or the promoter of the *PSA* gene in these cell lines is tissue specific. These possibilities were not studied further.

Among all androgenic compounds tested, only dihydroisoandrosterone sulphate, an inactive metabolite, was not able to stimulate PSA production. All other androgens were strong stimulators (Table 1). The physiological androgens testosterone and androsterone and their reduced forms dihydrotestosterone and dihydroandrosterone were able to induce PSA production at levels as low as $10^{-10}$ M and $10^{-11}$ M respectively. The lower active concentration of dihydrotestosterone (and dihydroandrosterone) is in accord with its higher affinity for the androgen receptor than testosterone (Levine, 1995). Strong stimulation was also observed with the synthetic compounds R1881 and R5020. In all cases tested, we observed a dose–response relationship. Although there is always a degree of cross-reactivity of steroid hormones with receptors other than the cognate receptors, the activity of androgens at levels around $10^{-11}$ M (a concentration 10- to 100-fold lower than the affinity constant of the testosterone – AR complex) strongly suggests that the effect is mediated through high-affinity binding to the androgen receptor and not through low-affinity binding to cross-reacting receptors. Among the four oestrogens tested, the three natural oestrogens – oestradiol, oestrone and oestriol – did not mediate any PSA production. These data suggest that the oestrogen receptor is not involved in *PSA* gene up-regulation in the breast carcinoma cell line T-47D.

17α-Ethinyloestradiol, a synthetic oestrogen, was a weak but consistent stimulator at concentrations ≥$10^{-8}$ M, suggesting that this steroid is not a pure oestrogen. Our data suggest that this steroid interacts with the androgen and/or the progesterone receptor leading to active complexes capable of weakly upregulating the *PSA* gene.

Among the group of glucocorticoids tested, the physiological glucocorticoid hydrocortisone and the synthetic glucocorticoid prednisone had no effect. The strong induction of betamethasone and dexamethasone and the weak induction by corticosterone (at concentrations ≥ $10^{-8}$ M), all of which have higher affinities for the glucocorticoid receptor than hydrocortisone and cortisone and do not bind to either AR or PR (Ojasoo et al, 1988), suggest that the glucocorticoid receptor is capable of regulating the *PSA* gene as well. The PSA stimulation, at high glucocorticoid concentrations only, may reflect the low concentration of this receptor in T-47D cells (Glover and Darbre, 1989; Nordeen et al, 1989).

With the exception of the inactive progesterone precursor 17α-hydroxyprogesterone, all other progesterone agonists tested were strong stimulators of PSA production. In dose–response experiments, we have shown that the three tested progestins were active at levels $10^{-10}$–$10^{-11}$ M. In particular, norgestimate, which exhibits highly specific high-affinity binding to the progesterone receptor than other progestin agonists (Phillips, 1990; Kafrissen, 1992; Phillips et al, 1992) and binds to androgen receptor very poorly, was active at levels down to $10^{-10}$ M. The data presented for the progestin agonists, in combination with data from blocking experiments (discussed below), strongly suggest that the progesterone receptor, activated by progestin, is capable of directly up-regulating the *PSA* gene.

In men, *PSA* gene regulation is under the control of testicular androgens through the androgen receptor. We speculate that in women, *PSA* gene regulation in organs such as the breast and the endometrium (Clements and Mukhtar, 1994) is mediated by progestins and androgens through the independent action of the progesterone and the androgen receptor.

Aldosterone, a natural mineralocorticoid, was capable of *PSA* regulation only weakly and at concentrations >$10^{-8}$ M.

Triamicinolone acetonide, a compound known to interact with the PR and GR but not the androgen receptor (Zava et al, 1979; Ojasoo et al, 1988) was found to strongly stimulate PSA production at concentrations as low as $10^{-10}$ M. This finding further strengthens our suggestion that the progesterone and glucocorticoid receptor can mediate PSA production without involvement of the AR.

Among the four antioestrogens, none was able to mediate PSA production consistent with the suggestion that the oestrogen receptor is not involved in *PSA* gene up-regulation. Among the group of antiandrogens, we observed some interesting phenomena. All these compounds bind to the androgen receptor leading to either inactive complexes (pure antiandrogens) or to complexes with some biological stimulatory activity (antiandrogens with weak agonist activity). In our system, hydroxyflutamide, casodex and nilutamide (anandron), which are known to bind to the androgen receptor with low affinity (<2% of testosterone affinity) (Teutsch et al, 1994), did not mediate any PSA production, suggesting formation of weak and inactive complexes with the androgen receptor. Cyproterone acetate, which binds to the androgen receptor with affinity approximately 10% of that of testosterone (Teutsch et al, 1994), was found to be a strong stimulator of PSA production, with activity even at concentrations of approximately $10^{-10}$ M. These data suggest that cyproterone acetate, a known antiandrogen that also interacts with the progesterone receptor (Teutsch et al., 1994; Levine, 1995) and has biological progestational activity (Levine, 1995), exerts its action on PSA regulation through binding to the progesterone receptor. Our finding that cyproterone acetate can up-regulate the *PSA* gene through the progesterone receptor in parallel to its expected down regulation of the *PSA* gene through androgen receptor blockade requires further investigation as monitoring PSA levels during cyproterone acetate treatment of prostate cancer may not be a reliable index of clinical response. Up-regulation of the *PSA* gene by cyproterone acetate through its progestational activity has not, to our knowledge, as yet been reported. It is currently unknown if this indeed happens in prostate cells in addition to breast cancer cell lines.

The newer antiandrogen RU56, 187 has affinity for the androgen receptor similar to testosterone but no detectable affinity for progesterone, glucocorticoid, mineralocorticoid or oestrogen receptors (Teutsch et al, 1994). In our system, we detected weak *PSA* gene up-regulation at RU56, 187 concentration ≥$10^{-8}$ M. This up-regulation strongly suggests that RU56,187 has weak androgen agonist activity. In this respect, our tissue culture system appears to be more sensitive than the in vitro systems used by Teutsch et al (1994) to evaluate RU56,187, concluding that this compound is totally devoid of binding to other steroid receptors and of any agonist effect. It remains to be determined if the weak agonist activity of RU56,187 has any biological significance.

Mifepristone (RU486, RU38,486) is a new antiprogestational agent with antiglucocorticoid and antiandrogenic activity. RU486 has been commercialized as an antiprogestin for first trimester pregnancy interruption. In our system, we found that Mifepristone has weak agonist activity, mediating *PSA* gene up-regulation at concentrations ≥$10^{-8}$ M. This agonist activity was not observed by Philibert et al (1985), further suggesting that their biological tests are not as sensitive as our tissue culture system in detecting such an effect. In support of our data are reports by others showing weak agonist activity of RU486 in various systems (Gravanis et al, 1985; Gronemeyer et al, 1991; Wehle et al, 1995). It remains to be seen if the weak agonist activity of mifepristone is mediated by the androgen, glucocorticoid or the progesterone receptor. Very weak agonist activity was also observed for the antiglucocorticoid cortexolone and the antimineralocorticoid spironolactone at concentrations ≥ $10^{-7}$ M.

While oestradiol has no positive effect on *PSA* gene regulation, blocking experiments have revealed that oestradiol could block the action of dihydrotestosterone and to a much lesser degree norgestrel but not norgestimate on *PSA* gene regulation (Table 2). There are two possible explanations for this phenomenon. First, high doses of oestradiol could cause its binding to the androgen receptor thus blocking the action of dihydrotestosterone (Ojasoo et al, 1988; Lea et al, 1989). Second, oestradiol would bind to the oestrogen receptor in T-47D cells and the active complex would further inhibit the action of active AR complexes. Active ER, AR and PR complexes compete for the same transcription factors including c-*jun* and c-*fos* as suggested previously (Pearce and Yamamoto, 1993). The fact that oestradiol blocks the stimulation by dihydrotestosterone but not the stimulation by norgestimate suggests that oestradiol blockade targets the AR but not the PR. Our finding of positive regulation of the *PSA* gene by androgen and progestin and the negative regulation by oestrogen suggests that PSA is regulated by a delicate balance between androgens, progestins and oestrogens.

 © Cancer Research Campaign 1997

Nilutamide and hydroxyflutamide, two antiandrogens that bind with low affinity to the androgen receptor, had moderate but not complete blocking activity (~30–40% on average) on dihydrotestosterone and an even lower blocking activity (≤20%) on norgestrel and no blocking activity on norgestimate. These data are expected as the stimulating steroids (e.g. dihydrotestosterone), having higher affinity for the androgen receptor, would displace a fraction of the blocker after they are added into the tissue culture system. On the other hand, RU56, 187, which has an affinity for the androgen receptor similar to testosterone, was able to block 85–91% of the activity of dihydrotestosterone. The lower blockade on norgestrel action (17–40% on average) and the absence of blockade on norgestimate action further suggests that a significant portion of norgestrel's and 100% of norgestimate's stimulation is mediated through the progesterone receptor to which RU56, 187 does not bind and could not block.

The antioestrogen ICI 182, 780 had little or no blocking effect on the stimulation of PSA production by dihydrotestosterone, norgestrel or norgestimate, in accordance with our view that the oestrogen receptor does not positively mediate PSA production in our system.

Mifepristone was an effective blocker of PSA production by dihydrotestosterone (70–80%) and an almost complete blocker of norgestrel and norgestimate (90–100%). This is in accord with our view that PSA production is mediated independently by the AR and the PR as mifepristone is known to block effectively the progesterone receptor and to a lesser but significant degree the androgen receptor (Philibert et al, 1985).

As expected, the antiglucocorticoid cortexolone and the antimineralocorticoid spironolactone had no effect on either dihydrotestosterone, norgestrel or norgestimate action as these two anti-hormones bind primarily to GR and MR and only with low affinity to other receptors that are involved in PSA production.

Taken together, our data suggest the following: the breast carcinoma cell line T-47D has the necessary receptors and other transcriptional machinery to produce PSA. Once stimulated by a steroid hormone, T-47D cells produce PSA mRNA within 1–2 h, synthesize detectable intracellular protein within 4 h and secrete detectable protein within 8 h. *PSA* gene regulation is under the control of androgens and progestins through the independent action of the androgen and progesterone receptors (positive regulation). Weak positive regulation may also be effected by high concentrations of glucocorticoids and mineralocorticoids. Oestrogens do not positively regulate the *PSA* gene but they act as blockers of androgen action. The most effective blockers of *PSA* gene regulation were found to be the anti-androgen RU56, 187 and the antiprogestin mifepristone. Our data, showing multihormone regulation of the *PSA* gene, are in accord with these of Glover and Darbe (1989) who concluded the same using T-47D cells transfected with the mammary tumour virus long terminal repeat sequences.

Our tissue culture system not only reproduces the phenomenon of PSA production by breast cancers but it also offers a means of testing the biological activity of candidate new hormonal and anti-hormonal agents. With this system, we have shown that two new anti-hormones, the antiandrogen RU56, 187 and the antiprogestin mifepristone, which were found to be completely devoid of agonist activity by traditional in vivo and in vitro techniques, have low but detectable androgen and/or progestin agonist activity demonstrated by their ability to upregulate the *PSA* gene.

Recently, we have obtained evidence that the progestin-mediated upregulation of the *PSA* gene occurs in vivo as well. We have reported PSA production by normal breast tissue in a female patient who was receiving an oral contraceptive containing norethidrone (Yu et al, 1995). PSA regulation by progestins in the prostate has not been reported but it is known that the prostate cells, in addition to AR, also contain PR (Mobbs and Liu, 1990). In another report, we described a patient who was receiving high doses of glucocorticoids and had an ovarian tumour producing PSA (Yu et al, 1995*c*).

Our data further supports the view that PSA may be regulated in diverse tissues containing PR. Tissues which contain PR include the breast (Yu et al, 1994*a*), the endometrium (Clements and Mukhtar, 1994), brain meningiomas (Carrol et al, 1993), blood vessel walls (Bergqvist et al, 1993), urinary tract (Pacchioni et al, 1992) and osteoclasts (Boivin et al, 1994). In view of these and other findings, we believe that it is time to study in detail the biological role of PSA in non-prostatic tissues. The presence of a significant prognostic value of this molecule in breast cancer (Yu et al, 1995*b*) suggests that this elegantly regulated enzyme may play some role in breast cancer initiation and progression.

### ACKNOWLEDGEMENTS

We would like to thank Debbie Tsuyuki and Herb Edgecomb for technical assistance, M Grynpas for the cell line SAOS and H Rocheford for the cell line BG-1. We would also like to thank D Peehl for providing us with hydroxyflutamide and Roussel and Zeneca for other compounds listed in the text.

### REFERENCES

Bergqvist A, Bergqvist D and Ferno M (1993) Estrogen and progesterone receptors in vessel walls: biochemical and immunological assays. *Acta Obstet Gynecol Scand* **72**: 10–16

Berthois Y, Katzenellenbogen JA and Katzenellenbogen BS (1986) Phenol red in tissue culture medium is a weak estrogen: implications concerning the study of estrogen responsive cells in culture. *Proc Natl Acad Sci USA* **83**: 2496–2500

Boivin G, Terrier-Anthoine C and Morel G (1994) Ultrastructural localization of endogenous hormones and receptors in bone tissue: an immunocytological approach in frozen samples. *Micron* **25**: 15–27

Carroll RS, Glowacka D, Dashner K and Black PM (1993) Progesterone receptors in meningiomas. *Cancer Res* **53**: 1312–1316

Clements A and Mukhtar A (1994) Glandular kallikriens and prostate specific antigen are expressed in the human endometrium. *J Clin Endocrinol Metab* **78**: 1536–1539

Deguchi T, Dio T, Ehara H, ITO S, Takahashi Y, Nishino Y, Fujihiro S, Kawamura T, Komeda H, Horie M, Kaji H, Shimokawa K, Tanaka T and Kawada Y (1993) Detection of micrometastatic prostate cancer cells in lymph nodes by reverse transcriptase-polymerase chain reaction. *Cancer Res* **53**: 5350–5354

Diamandis EP and Yu H (1995) New biological functions of prostate specific antigen? *J Clin Endocrinol Metab* **80**: 1515–1517

Digby M, Zhang XY and Richards RI (1989) Human prostate specific antigen (PSA) gene: structure and linkage to the kallikrein-like gene, hGK-1. *Nucleic Acids Res* **17**, 2137

Ferguson RA, Yu H, Kalyvas M, Zammit S and Diamandis EP (1996) Ultrasensitive detection of prostate specific antigen by a new time resolved immunofluorometric assay and the Immulite immunochemiluminescent third generation assay: potential applications in prostate and breast cancers. *Clin Chem*, **42**: 675–684

Glover JF and Darbre FD (1989) Multihormone regulation of MMTV-LTR in transfected T-47D human breast cancers. *J Steroid Biochem* **32**: 357–363

Gravanis A, Schaison G, George M, Debrux J, Satyaswaroop PG, Baulieu EE and Robel P (1985) Endometrial and pituitary responses to the steroidal antiprogestin RU 486 in postmenopausal women. *J Clin Endocrinol Metab*, **60**: 156–163

© Cancer Research Campaign 1997

Gronemeyer H, Meyer M-E, Bocquel M-T, Kastner P, Turcotte B and Chambon P (1991) Progestin receptors: isoforms and antihormone action. *J Steroid Biochem Mol Biol* **40**: 271–278

Henttu P and Vihko P (1989) cDNA coding for the entire human prostate specific antigen shows high homologies to the human tissue kallikrein genes. *Biochem Biophys Res Commun* **160**: 903–910

Henttu P, Liao S and Vihko P (1992) Androgens up-regulate the human prostate-specific antigen messenger ribonucleic acid (mRNA), but down-regulate the prostatic acid phosphatase mRNA in the LNCaP cell line. *Endocrinology* **130**: 766–772

Kafrissen ME (1992) A norgestimate-containing oral contraceptive: review of clinical studies. *Am J Obstet Gynecol* **167**: 1196–1202

Klobeck HG, Combriato G, Schulz P, Arbuson V and Fittler F (1989) Genomic sequence of human prostate specific antigen (PSA). *Nucleic Acids Res* **17**: 3981

Lea OA, Kuinnsland S and Thorsen T (1989) Improved measurement of androgen receptors in human breast cancer. *Cancer Res* **49**: 7162–7167

Levine AC (1995) Pathogenesis and medical management of benign prostatic hyperplasia. *Trends Endocrinol Metab* **6**: 128–132

Luke MC and Coffey DS (1994) Human androgen receptor binding to the androgen response element of prostate specific antigen. *J Androl* **15**: 41–51

Lundwall A (1989) Characterization of the gene for prostate specific antigen, a human glandular kallikrein. *Biochem Biophys Res Commun* **161**: 1151–1159

Lundwall A and Lilja H (1987) Molecular cloning of human prostate specific antigen cDNA. *Febs Lett*, **214**: 317–322

Mobbs BG and Liu Y (1990) Immunohistochemical localization of progesterone receptor in benign and malignant human prostate. *Prostate* **16**: 245–251

Murtha P, Tindall DJ and Young CYF (1993) Androgen induction of a human prostate specific kallidrein, hKLK2: characterization of an androgen response element in the 5′ promoter region of the gene. *Biochemistry* **32**: 6459–6464

Nordeen SK, Kuhnel B, Lawler-Heavner J, Barber DA and Edwards DP (1989) A quantitative comparison of dual control of hormone response element by progestins and glucocorticoids in the same cell line. *Mol Endocrinol* **3**: 1270–1278

Ojasso T, Dore JC, Gilbert J and Raynaud JP (1988) Binding of steroids to the progestin and glucocorticoid receptors analyzed by correspondence analysis. *J Med Chem* **31**: 1160–1169

Okazaki T (1992) Detection of estrogen receptor (ER) mRNA by use of reverse transcriptase-polymerase chain reaction (RT-PCR) assay; comparison with dextran coated charcol (DCC) assay and immunocytochemical assay. *Jpn J Cancer Chemother* **19**: 361–366

Pacchioni D, Revelli A, Casetta G, Cassoni P, Piana P, Tizzani A, Buscati G and Massobrio M (1992) Immunochemical detection of estrogen and progesterone receptors in the normal urinary bladder and in pseudomembranous trigonitis. *J Endocrinol Invest* **15**: 719–725

Pearce D and Yamamoto KR (1993) Mineralocorticoid and glucocorticoid receptor activities distinguished by nonreceptor factors at composite response element. *Science* **259**: 1161–1165

Philibert D, Moguilewsky M, Mary I, Lecaque D, Tournemine C, Secchi J and Deraedt R (1985) Pharmacological profile of RU 486 in animals. In *The Antiprogestin Steroid RU 486 and Human Fertility Control*, Beaulieu EE and Segal SJ (eds), pp. 49–68. Plenum Publishing: New York

Phillips A (1990) The selectivity of a new progestin. *Acta Obstet Gynecol Scand* **152** (Suppl): 21–24

Phillips A, Hahn DW and McGuire JL (1992) Preclinical evaluation of norgestimate, a progestin with minimal androgenic activity. *Am J Obstet Gynecol* **167**: 1191–1196

Riegman PH, Klaassen P, Van Der Korput JA, Romijn JC and Trapman J (1988) Molecular cloning and characterization of novel prostate antigen cDNA's. *Biochem Biophys Res Commun* **155**: 181–188

Riegman PH, Vliestra RJ, Van Der Korput Jagm, Brinkman AO and Trapman J (1991) The promoter of the prostate specific antigen gene contains a functional androgen responsive element. *Mol Endocrinol* **5**: 1921–1930

Schulz P, Stucka R, Feldmann H, Combriato G, Klobeck HG and Fittler, F (1988) Sequence of a cDNA clone encompassing the complete mature human prostate specific antigen (PSA) and an unspliced leader sequence. *Nucleic Acids Res* **16**: 6226

Smith MR, Biggar S and Hussain M (1995) Prostate-specific antigen messenger RNA is expressed in non-prostate cells: implications for detection of micrometastases. *Cancer Res* **55**: 2640–2644

Teutsch G, Goubet F, Battmann T, Bonfils A, Bouchoux F, Cerede E, Gofflo D, Gaillard-Kelly M and Philibert D (1994) Non-steroidal antiandrogens: synthesis and biological profile of high-affinity ligands for the androgen receptor. *J Steroid Biochem Mol Biol* **48**: 111–119

Wehle H, Moll J and Cato ACB (1995) Molecular identification of steroid analogs with dissociated antiprogestin activities. *Steroids* **60**: 368–374

Yu H, Diamandis EP and Sutherland DJA (1994*a*) Immunoreactive prostate specific antigen levels in female and male breast tumors and its association with steroid hormone receptors and patient age. *Clin Biochem* **27**: 75–79

Yu H, Diamandis EP, Zarghami N and Grass L (1994*b*) Induction of prostate specific antigen production by steroids and tamoxifen in breast cancer cell lines. *Breast Cancer Res Treat* **32**: 291–300

Yu H, Diamandis EP, Monne M and Croce CM (1995*a*) Oral contraceptive-induced expression of prostate specific antigen in the female breast. *J Biol Chem* **270**: 6615–6618

Yu H, Giai M, Diamandis EP, Katsaros D, Sutherland DJA, Levesque MA, Roagna R, Ponzone R and Sismondi P (1995*b*) Prostate specific antigen is a new favourable prognostic indicator for women with breast cancer. *Cancer Res* **55**: 2104–2110

Yu H, Diamandis EP, Levesque M, ASA S, Monne M and Croce CM (1995*c*) Expression of the prostate-specific antigen gene by a primary ovarian carcinoma. *Cancer Res* **55**: 1603–1606

Zava DT, Landrum B, Horwitz KB and Mcguire WL (1979) Androgen receptor assay with [$^3$H]methyltrienolone (R1881) in the presence of progesterone receptors. *Endocrinology* **104**: 1007–1012

*© Cancer Research Campaign 1997*


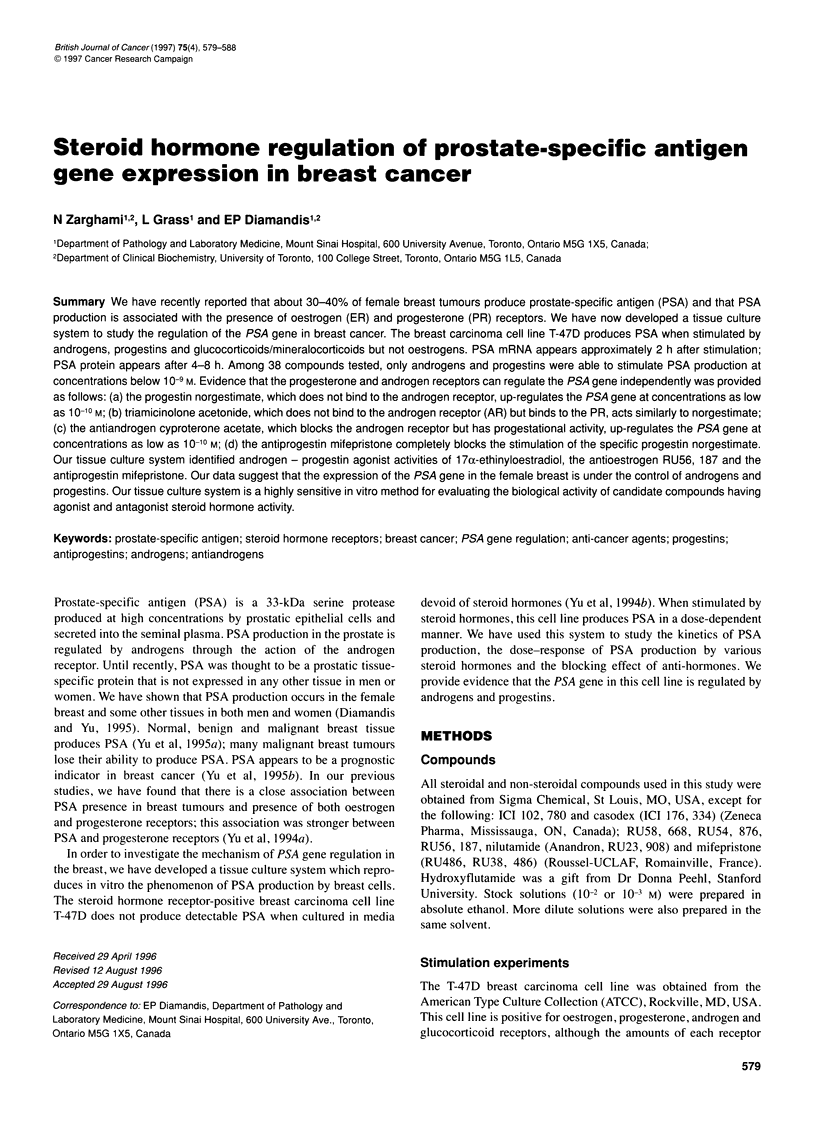

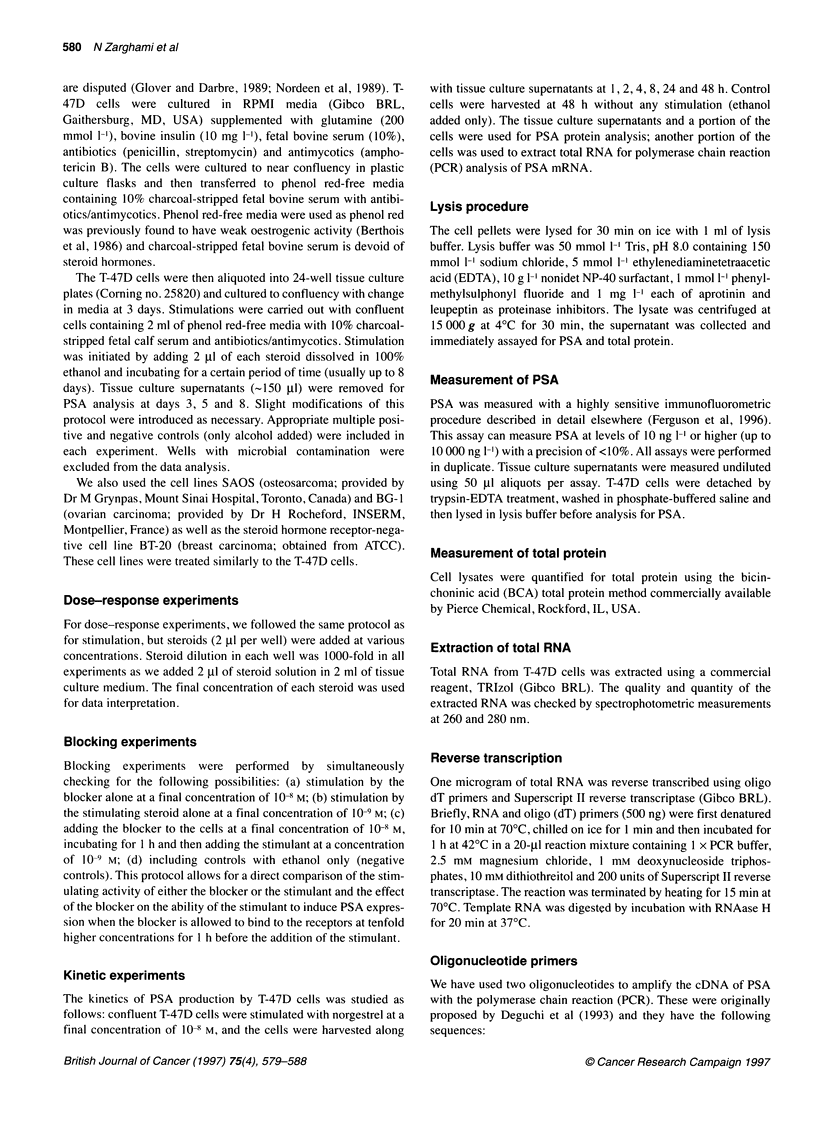

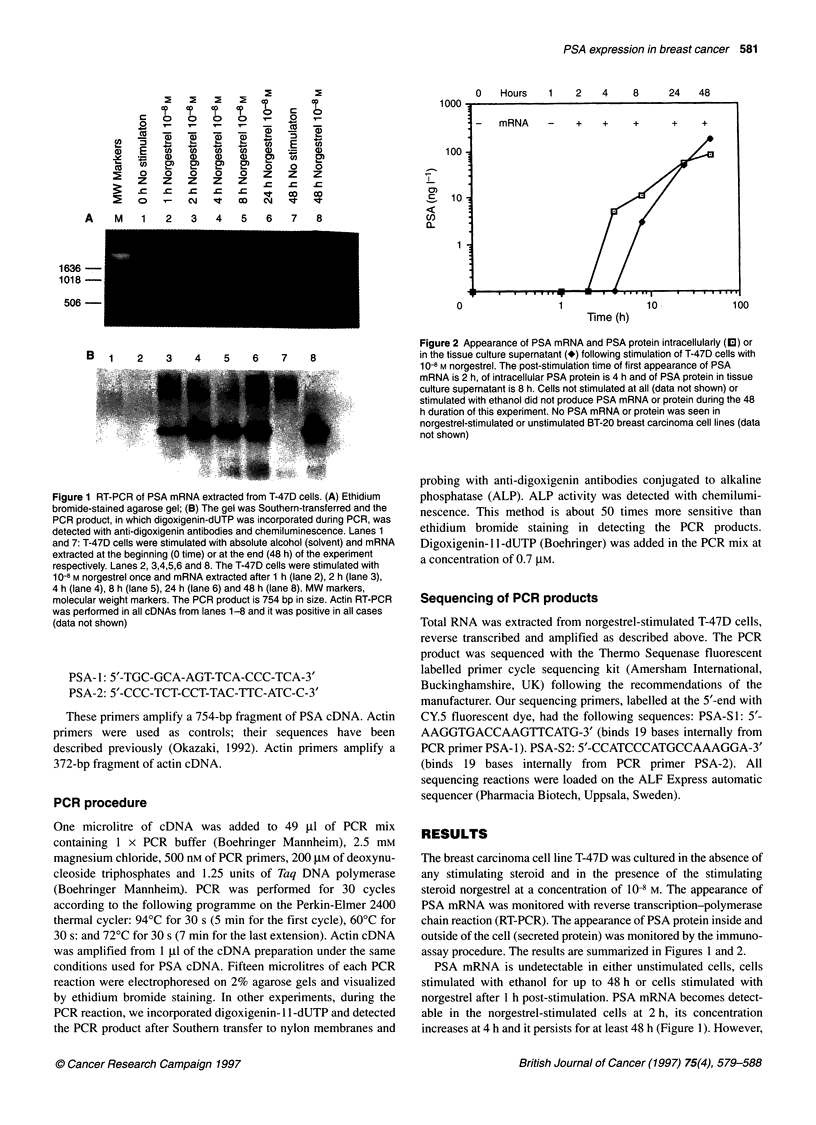

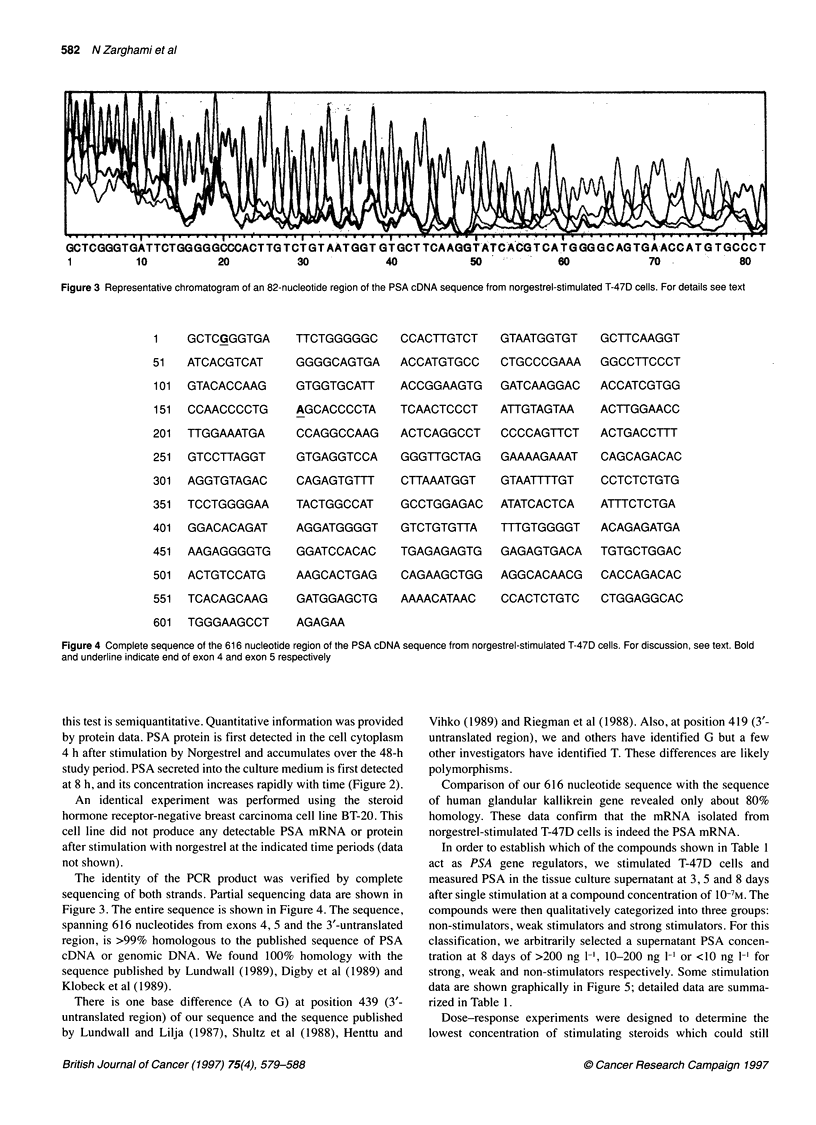

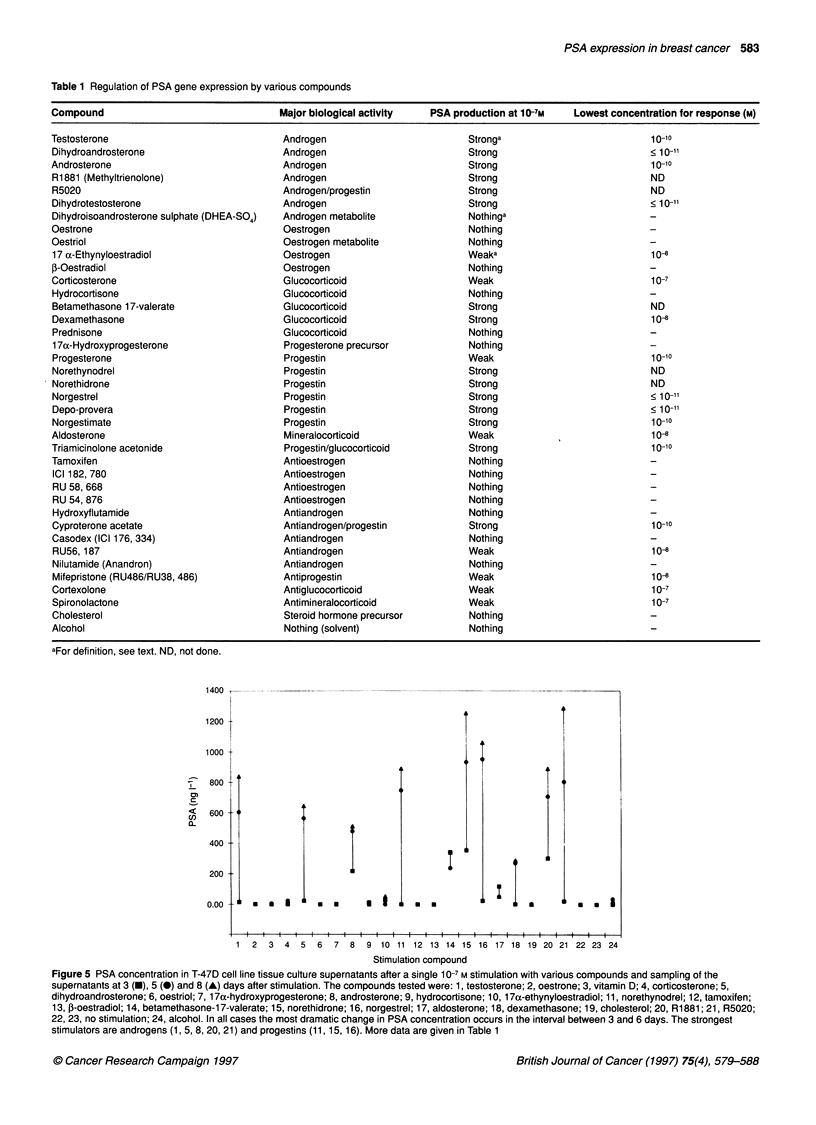

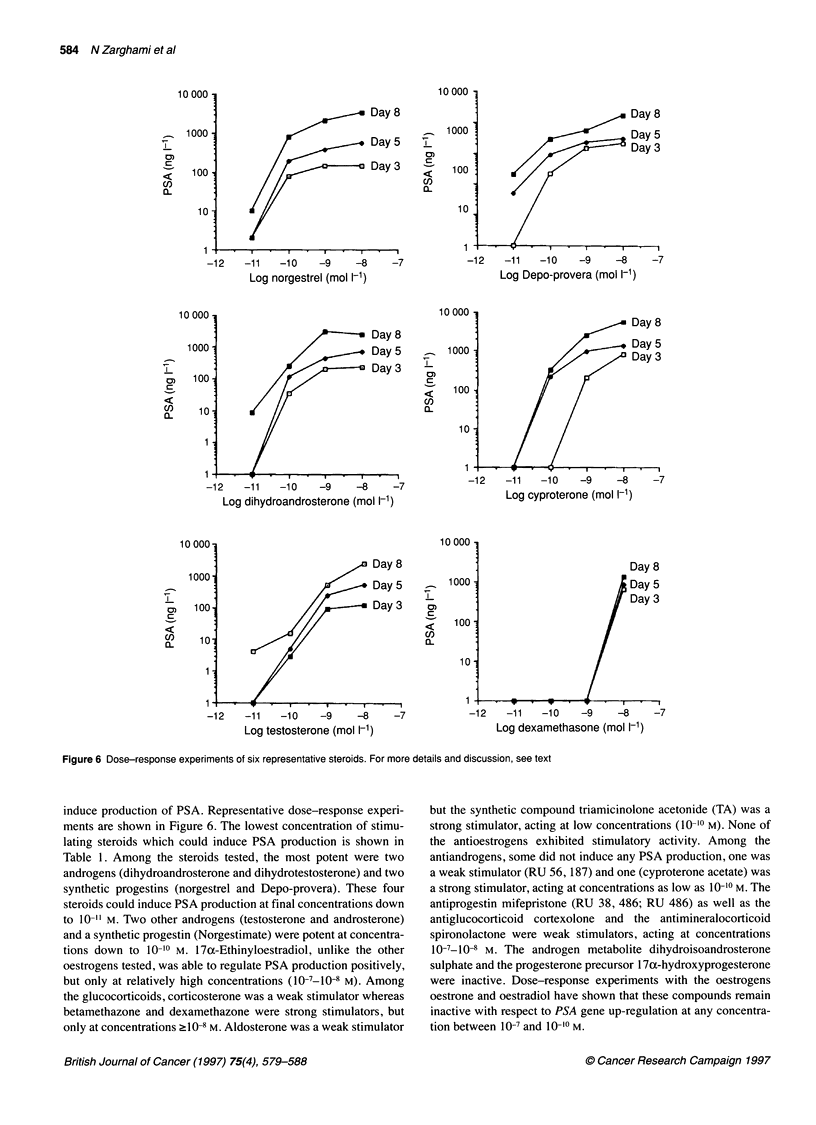

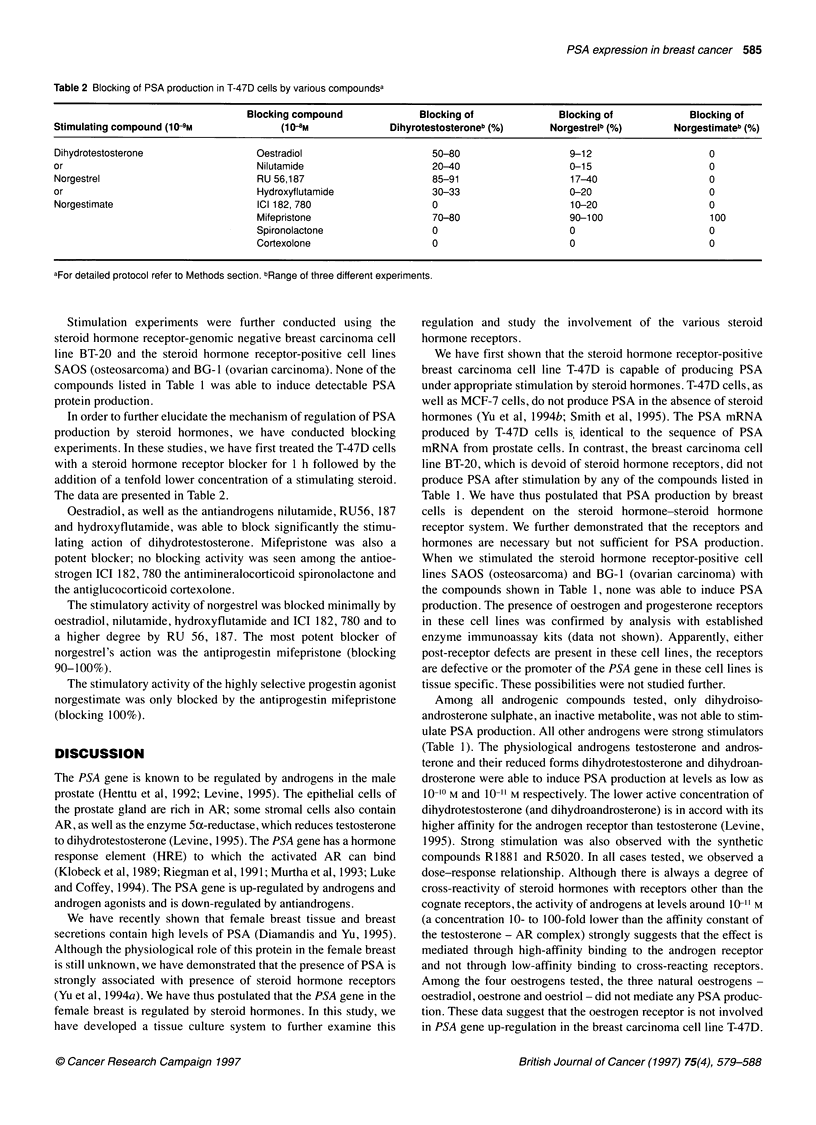

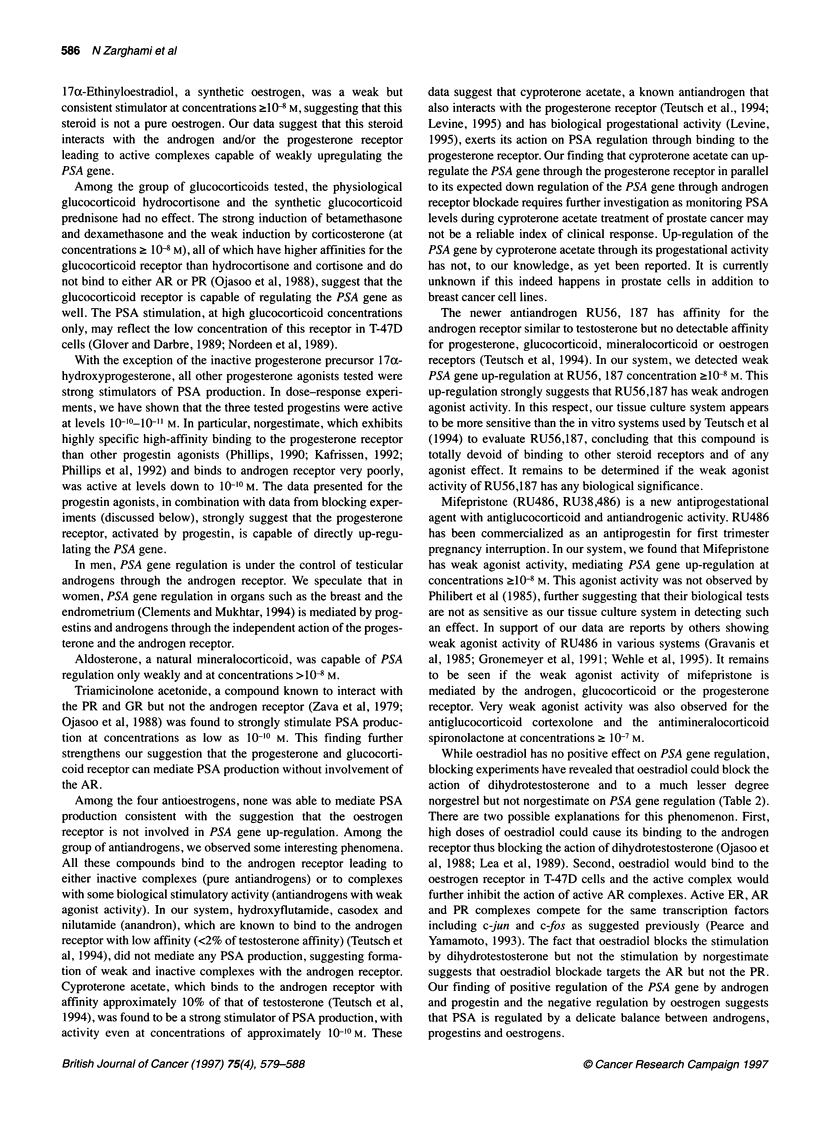

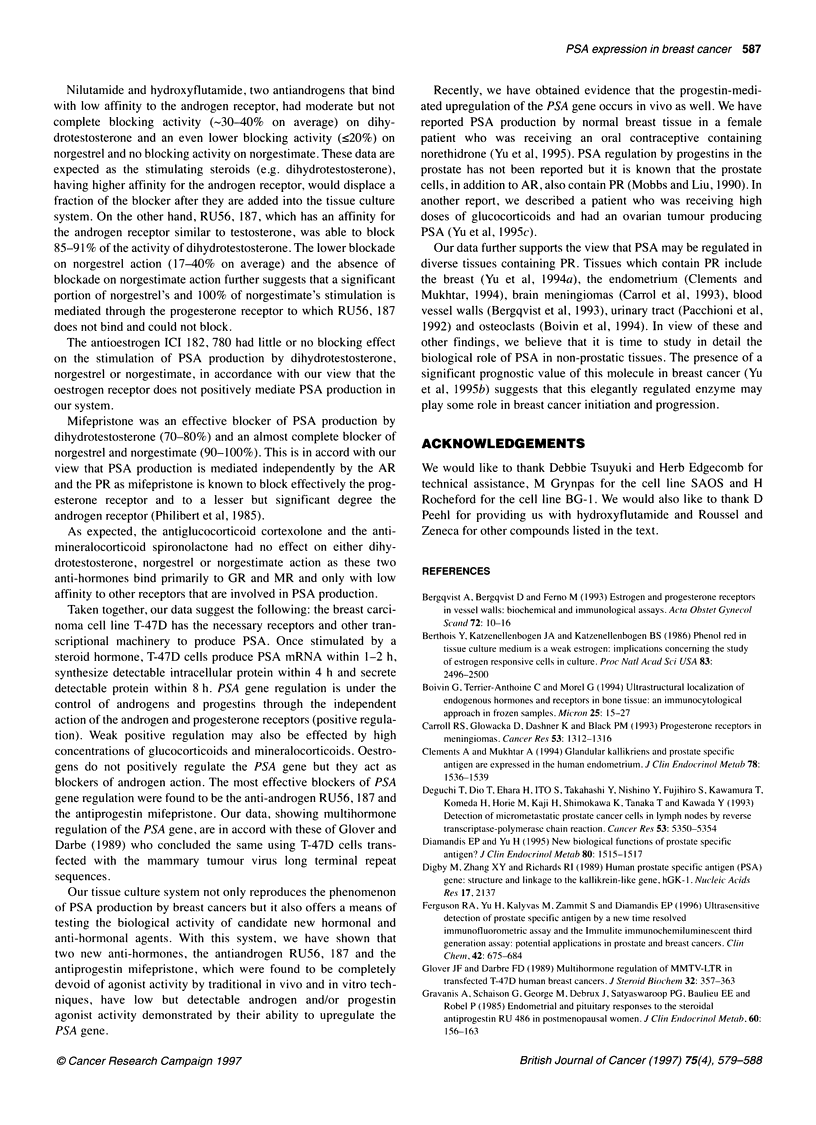

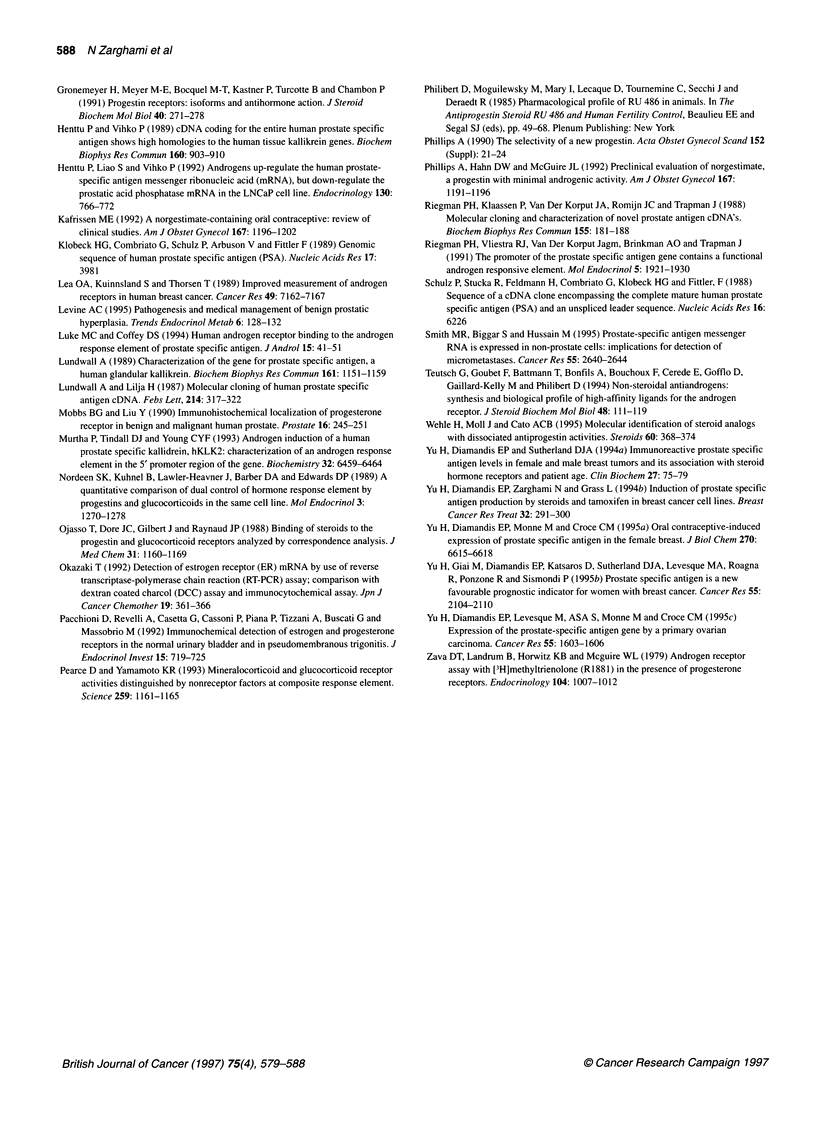


## References

[OCR_01120] Bergqvist A., Bergqvist D., Fernö M. (1993). Estrogen and progesterone receptors in vessel walls. Biochemical and immunochemical assays.. Acta Obstet Gynecol Scand.

[OCR_01125] Berthois Y., Katzenellenbogen J. A., Katzenellenbogen B. S. (1986). Phenol red in tissue culture media is a weak estrogen: implications concerning the study of estrogen-responsive cells in culture.. Proc Natl Acad Sci U S A.

[OCR_01131] Boivin G., Anthoine-Terrier C., Morel G. (1994). Ultrastructural localization of endogenous hormones and receptors in bone tissue: an immunocytological approach in frozen samples.. Micron.

[OCR_01136] Carroll R. S., Glowacka D., Dashner K., Black P. M. (1993). Progesterone receptor expression in meningiomas.. Cancer Res.

[OCR_01151] Diamandis E. P., Yu H. (1995). New biological functions of prostate-specific antigen?. J Clin Endocrinol Metab.

[OCR_01155] Digby M., Zhang X. Y., Richards R. I. (1989). Human prostate specific antigen (PSA) gene: structure and linkage to the kallikrein-like gene, hGK-1.. Nucleic Acids Res.

[OCR_01160] Ferguson R. A., Yu H., Kalyvas M., Zammit S., Diamandis E. P. (1996). Ultrasensitive detection of prostate-specific antigen by a time-resolved immunofluorometric assay and the Immulite immunochemiluminescent third-generation assay: potential applications in prostate and breast cancers.. Clin Chem.

[OCR_01168] Glover J. F., Darbre P. D. (1989). Multihormone regulation of MMTV-LTR in transfected T-47-D human breast cancer cells.. J Steroid Biochem.

[OCR_01174] Gravanis A., Schaison G., George M., de Brux J., Satyaswaroop P. G., Baulieu E. E., Robel P. (1985). Endometrial and pituitary responses to the steroidal antiprogestin RU 486 in postmenopausal women.. J Clin Endocrinol Metab.

[OCR_01183] Gronemeyer H., Meyer M. E., Bocquel M. T., Kastner P., Turcotte B., Chambon P. (1991). Progestin receptors: isoforms and antihormone action.. J Steroid Biochem Mol Biol.

[OCR_01193] Henttu P., Liao S. S., Vihko P. (1992). Androgens up-regulate the human prostate-specific antigen messenger ribonucleic acid (mRNA), but down-regulate the prostatic acid phosphatase mRNA in the LNCaP cell line.. Endocrinology.

[OCR_01188] Henttu P., Vihko P. (1989). cDNA coding for the entire human prostate specific antigen shows high homologies to the human tissue kallikrein genes.. Biochem Biophys Res Commun.

[OCR_01200] Kafrissen M. E. (1992). A norgestimate-containing oral contraceptive: review of clinical studies.. Am J Obstet Gynecol.

[OCR_01209] Lea O. A., Kvinnsland S., Thorsen T. (1989). Improved measurement of androgen receptors in human breast cancer.. Cancer Res.

[OCR_01217] Luke M. C., Coffey D. S. (1994). Human androgen receptor binding to the androgen response element of prostate specific antigen.. J Androl.

[OCR_01221] Lundwall A. (1989). Characterization of the gene for prostate-specific antigen, a human glandular kallikrein.. Biochem Biophys Res Commun.

[OCR_01224] Lundwall A., Lilja H. (1987). Molecular cloning of human prostate specific antigen cDNA.. FEBS Lett.

[OCR_01228] Mobbs B. G., Liu Y. (1990). Immunohistochemical localization of progesterone receptor in benign and malignant human prostate.. Prostate.

[OCR_01231] Murtha P., Tindall D. J., Young C. Y. (1993). Androgen induction of a human prostate-specific kallikrein, hKLK2: characterization of an androgen response element in the 5' promoter region of the gene.. Biochemistry.

[OCR_01235] Nordeen S. K., Kühnel B., Lawler-Heavner J., Barber D. A., Edwards D. P. (1989). A quantitative comparison of dual control of a hormone response element by progestins and glucocorticoids in the same cell line.. Mol Endocrinol.

[OCR_01241] Ojasoo T., Doré J. C., Gilbert J., Raynaud J. P. (1988). Binding of steroids to the progestin and glucocorticoid receptors analyzed by correspondence analysis.. J Med Chem.

[OCR_01252] Pacchioni D., Revelli A., Casetta G., Cassoni P., Piana P., Tizzani A., Bussolati G., Massobrio M. (1992). Immunohistochemical detection of estrogen and progesterone receptors in the normal urinary bladder and in pseudomembranous trigonitis.. J Endocrinol Invest.

[OCR_01258] Pearce D., Yamamoto K. R. (1993). Mineralocorticoid and glucocorticoid receptor activities distinguished by nonreceptor factors at a composite response element.. Science.

[OCR_01274] Phillips A., Hahn D. W., McGuire J. L. (1992). Preclinical evaluation of norgestimate, a progestin with minimal androgenic activity.. Am J Obstet Gynecol.

[OCR_01270] Phillips A. (1990). The selectivity of a new progestin.. Acta Obstet Gynecol Scand Suppl.

[OCR_01279] Riegman P. H., Klaassen P., van der Korput J. A., Romijn J. C., Trapman J. (1988). Molecular cloning and characterization of novel prostate antigen cDNA's.. Biochem Biophys Res Commun.

[OCR_01284] Riegman P. H., Vlietstra R. J., van der Korput J. A., Brinkmann A. O., Trapman J. (1991). The promoter of the prostate-specific antigen gene contains a functional androgen responsive element.. Mol Endocrinol.

[OCR_01289] Schulz P., Stucka R., Feldmann H., Combriato G., Klobeck H. G., Fittler F. (1988). Sequence of a cDNA clone encompassing the complete mature human prostate specific antigen (PSA) and an unspliced leader sequence.. Nucleic Acids Res.

[OCR_01295] Smith M. R., Biggar S., Hussain M. (1995). Prostate-specific antigen messenger RNA is expressed in non-prostate cells: implications for detection of micrometastases.. Cancer Res.

[OCR_01300] Teutsch G., Goubet F., Battmann T., Bonfils A., Bouchoux F., Cerede E., Gofflo D., Gaillard-Kelly M., Philibert D. (1994). Non-steroidal antiandrogens: synthesis and biological profile of high-affinity ligands for the androgen receptor.. J Steroid Biochem Mol Biol.

[OCR_01307] Wehle H., Moll J., Cato A. C. (1995). Molecular identification of steroid analogs with dissociated antiprogestin activities.. Steroids.

[OCR_01333] Yu H., Diamandis E. P., Levesque M., Asa S. L., Monne M., Croce C. M. (1995). Expression of the prostate-specific antigen gene by a primary ovarian carcinoma.. Cancer Res.

[OCR_01321] Yu H., Diamandis E. P., Monne M., Croce C. M. (1995). Oral contraceptive-induced expression of prostate-specific antigen in the female breast.. J Biol Chem.

[OCR_01311] Yu H., Diamandis E. P., Sutherland D. J. (1994). Immunoreactive prostate-specific antigen levels in female and male breast tumors and its association with steroid hormone receptors and patient age.. Clin Biochem.

[OCR_01316] Yu H., Diamandis E. P., Zarghami N., Grass L. (1994). Induction of prostate specific antigen production by steroids and tamoxifen in breast cancer cell lines.. Breast Cancer Res Treat.

[OCR_01326] Yu H., Giai M., Diamandis E. P., Katsaros D., Sutherland D. J., Levesque M. A., Roagna R., Ponzone R., Sismondi P. (1995). Prostate-specific antigen is a new favorable prognostic indicator for women with breast cancer.. Cancer Res.

[OCR_01338] Zava D. T., Landrum B., Horwitz K. B., McGuire W. L. (1979). Androgen receptor assay with [3H]methyltrienolone (R1881) in the presence of progesterone receptors.. Endocrinology.

